# Factors associated with road traffic injury severity among victims retrieved by pre-hospital emergency services in Rwanda

**DOI:** 10.12688/f1000research.172231.1

**Published:** 2025-11-19

**Authors:** Eric Uwitonze, Emmanuel Biracyaza

**Affiliations:** 1 Department of Epidemiology and Biostatistics, College of Medicine and Health Sciences Huye, University of Rwanda, Butare, Southern Province, Rwanda; 2 Emergency Medical Service (EMS) Division, Rwanda Biomedical Centre, Kigali, Rwanda; 3 School of Rehabilitation, Universite de Montreal, Montreal, Québec, Canada; 4 Centre for Interdisciplinary Research in Rehabilitation of Greater Montréal, Montréal, Canada

**Keywords:** Road Traffic Injuries; Emergency care; Severe injury; Predictors; Public health

## Abstract

**Background:**

Traumatic injuries remain critical public health concerns, placing psychosocial and economic burdens on individuals, families, and healthcare systems. Despite being a leading cause of morbidity and mortality globally, especially in low-and middle-income countries, few studies have examined predictors of injury severity in pre-hospital settings. Most research focuses on injury incidence, with limited attention to pre-hospital factors. We aimed assessing the prevalence of severe injuries and their associated factors among patients managed by pre-hospital emergency services.

**Methods:**

A cross-sectional study was utilized medical registry data from 1,162 RTI victims. Demographic, epidemiological, and clinical information were collected, with injuries categorized as severe or non-severe based on the Injury Severity Score. Bivariate and multivariable logistic regression models were conducted to indicate associated factors of severe injury.

**Results:**

Among 1162 victims, 165 (14%) experienced severe injury. Our results showed that females were less likely to experience severe injury (aOR=0.47, 95%CI:0.26–0.79) than males. Regarding trauma mechanism, car-to-pedestrian collisions (aOR=2.3, 95%CI:1.25–4.1), car-to-motorcycle collisions considerably increased the likelihoods of severe injury (aOR=3.88, 95%CI:1.16–13.05) compared to car-only crashes. Alcohol users were more likely to experience severe injury (aOR=3.37; 95%CI: 2.04-5.56) than non-users. Those who travelled distance ranged 21-40 km had higher likelihoods (aOR=2.91, 95%CI:1.27–6.63), while those with more than 40 km faced higher likelihoods of severe injury (aOR=2.64, 95% CI:1.11–6.25) than individuals with less than 20 km to reach to a healthcare facility. Those with extremity injuries (aOR=0.28, 95% CI:0.15–0.52), chest injuries (aOR=0.40, 95%CI:0.23–0.71, p=.002) had lower likelihoods of severe injury than those with head trauma.

**Conclusion:**

This study provides valuable insights into the factors influencing injury severity in the pre-hospital setting. The findings underscore the importance of strengthening early identification and rapid stabilization of high-risk patients during pre-hospital care. Future research using prospective longitudinal designs is recommended to confirm causality.

## Background

Road traffic injuries (RTIs) represent a major public health and socio-economic challenge worldwide, responsible for approximately 1.19 million deaths annually in 2021, ^1^ a decrease from 1.35 million in 2016. ^2– 4^ Nearly 90% of these fatalities occur in low- and middle-income countries (LMICs), where road safety systems remain underdeveloped. RTIs are now the eighth leading cause of death globally and a major contributor to disability-adjusted life years (DALYs) lost. ^5, 6^ More than half of these issues involve vulnerable road users, including pedestrians, cyclists, and motorcyclists. ^7^ Without stronger prevention measures, the World Health Organization projects a 40% increase in global RTIs, particularly in LMICs, with RTIs expected to become the fifth leading cause of death by 2030. ^6, 8, 9^

Although high-income countries have made significant progress in reducing RTI fatalities, ^10^ low- and middle-income countries (LMICs) continue to face disproportionately high rates of traffic crashes and related deaths. Several contributing factors have been identified, including rapid motorization, substandard road infrastructure, excessive speeding, weak enforcement of traffic regulations, limited access to trauma care, and insufficient pre-hospital emergency services. ^11– 13^ Individual-level factors such as being male, young age, and low educational attainment are also associated with increased injury severity, along with clinical factors such as multiple injuries, head, neck, or spinal trauma, and the victim’s role in traffic (driver, passenger, or pedestrian). Additional environmental and contextual risks include long travel distances to healthcare facilities, seasonal hazards like rain, and lack of protective gear. Characteristics of the crash itself such as location, type, time of occurrence, and absence of safety equipment (e.g., helmets, seat belts, airbags) also play a role. ^14– 18^ Furthermore, behavioral factors, particularly alcohol consumption, and underlying health conditions, such as chronic illnesses (e.g., diabetes), have been linked to the likelihood of sustaining severe injuries. ^12, 19– 21^

Like other LMICs, Rwanda faces a growing burden of RTIs, which pose a significant challenge to both public health and economic development. ^22^ Data from the Rwanda National Police and Ministry of Infrastructure identify road traffic crashes as one of the leading causes of injury-related deaths and hospitalizations. ^23, 24^ Recent studies show that motorcyclists and pedestrians are disproportionately affected, and thousands of Rwandans suffer severe trauma from road crashes every year. The resulting health consequences translate into a substantial DALY burden and overwhelm Rwanda’s already stretched healthcare system. ^22^

Despite national efforts to improve road safety in Rwanda, ^23– 25^ critical gaps persist particularly in pre-hospital emergency care, road infrastructure, and strategies addressing the root causes of road RTIs. These shortcomings continue to undermine progress and contribute to avoidable injuries and deaths. There is limited evidence in Rwanda and similar settings regarding predictors of injury severity in the pre-hospital context, which is crucial for improving early trauma care and outcomes. Given this gap, this study aimed to assess the prevalence of severe injuries and determine their associated factors among RTI victims managed by pre-hospital emergency services in Rwanda. The findings are expected to support national policy reforms, improve emergency response systems, and inform targeted, evidence-based injury prevention strategies ultimately contributing to reduced mortality and improved trauma care from the point of first contact.

## Methods and materials

### Study design

This study employed a retrospective cross-sectional design using secondary data from the registries of Emergency Medical Services (EMS), known as Service d’Aide Médicale d’Urgence (SAMU). It encompassed all patients attended by pre-hospital emergency ambulance services.

### Study setting and data source

Rwanda, a landlocked country in East Africa, has a population of approximately 14 million, with a majority being young people. It is one of the most densely populated nations in Africa, covering an area of 26,338km ^2^. ^26^ The study was conducted within the Service d’Aide Médicale Urgente (SAMU), the national pre-hospital emergency care division of the Ministry of Health in Rwanda. SAMU operates in Kigali, capital city of Rwanda and provides emergency transportation and pre-hospital care for trauma victims, including those injured in RTIs. Patient information is systematically documented in a computerized registry developed in partnership with Virginia Commonwealth University, which captures demographic and clinical data relevant to emergency medical care. ^27^ This institution has a diverse team of healthcare professionals, including nurses and anesthetists, all of whom receive training in emergency care and first aid to ensure the delivery of high-quality services. Depending on the severity of the case, their main services also include patient transportation, pre-hospital care, first aid, and client transfer. The drivers, who are also trained in first aid, play an important role by providing safe, timely, and high-quality support during patient transport. ^28^

### Participants, sampling, and eligibility criteria

We conducted this research using the SAMU pre-hospital registry to include all patients whose primary injury mechanism was a RTI between January 1 and December 31, 2020. Eligible cases included individuals injured as motor vehicle occupants, motorcyclists, bicyclists, pedestrians, or users of animal-drawn carts on roads. From 2,062 identified cases, 900 were excluded due to missing demographic or clinical data, resulting in a final analytic sample of 1,162 patients (56.5%). Inclusion criteria required that the registry listed RTI as the main cause of injury and that patient records contained complete demographic and clinical information; cases with injuries unrelated to RTIs or with incomplete data were excluded. Furthermore, a census sampling approach was used including all eligible cases within the study period to provide more comprehensive coverage of the target population and minimize sampling bias.

### Study variables

The study variables in this study were categorized as dependent and independent. The dependent variable was injury severity, classified using the Injury Severity Score (ISS). This ISS was used as an anatomical scoring system that provide overall scores for patients with multiple injuries. ^29^ The ISS is derived from the Abbreviated Injury Scale (AIS) by selecting the three most severely injured body regions, squaring the AIS scores for these regions, and summing them to produce a total score ranging from 0 to 75. ^30^ In this research, injury severity was dichotomized as either severe (ISS ≥ 9) or non-severe (ISS <9). ^31^ This cutoff was chosen based on established thresholds in trauma research, where an ISS of 9 or higher is commonly used to indicate clinically significant injuries requiring advanced medical attention.

The independent variables included a range of socio-demographic factors (e.g., age, gender, and health insurance status), clinical or health-related factors (e.g., primary complaints, trauma mechanism, time of collision), behavioral factors such as alcohol use, and environmental factors including distance from the accident site to the health facility. These variables were analyzed to explore their association with injury severity. The head injury or traumatic brain injury (TBI) measured using Glasgow Coma Scale (GCS) to indicate the level of consciousness. While this instrument show severity of brain injury, we categorised the patients who experienced head injury as severe (≤8) TBI or non-severe injury (>8). In addition, the classification of variables such as trauma mechanism and time of collision was guided by the structure of EMS records and prior studies conducted in sub-Saharan Africa. ^11, 32, 33^ These elements are already standardized within the EMS reporting system, ensuring consistency in data extraction and analysis. While previous studies did not categorize distance to health facilities, we created three distance-based categories to reflect access challenges. Distances over 5 km were considered significant due to their potential to delay care, aligning with national goals to improve timely access to emergency services. ^34, 35^

### Data collection and materials

Data was collected from SAMU’s electronic data registry between July 13 and November 30, 2022. SAMU staff collected demographic and clinical data using standardized client forms, which were later entered into an electronic registry. Data collection was supervised by SAMU’s pre-hospital team leader and overseen by the study’s primary author. Enumerators received two days of training on the data collection process to ensure accuracy. Data from the electronic registry was exported to the Statistical Package for the Social Sciences (SPSS) software, version 28, for statistical analysis.

### Data analysis

Following data cleaning and the removal of incomplete variables, the analysis was conducted in two stages: descriptive and analytical. In the descriptive analysis, statistical parameters such as mean, standard deviation, frequency, and percentage were used to summarize the data. Cross-tabulations were performed to explore the distribution of injury severity across the independent variables. For the analytical phase, bivariate logistic regression was performed to calculate crude odds ratios (cORs) and assess associations between injury severity and each independent variable. Variables found to be statistically significant in the bivariate analysis were included in multivariable logistic regression models using a forward selection method. This approach allowed for the calculation of adjusted odds ratios (aOR) with 95% confidence intervals (CI) for each significant variable, identifying factors associated with severe injury. To address potential multicollinearity, the variance of inflation factor (VIF) was assessed for all independent variables. A VIF threshold of 5 was used; variables with VIF values exceeding this cutoff were considered to exhibit high multicollinearity and were subsequently reviewed or excluded from the model to ensure robustness. ^36, 37^

### Ethics

This research did not involve direct interaction with human participants or the collection of new data from individuals. Instead, it employed secondary data obtained from existing medical records and laboratory logbooks. Ethical approval for the study was obtained from the Institutional Review Board of the College of Medicine and Health Sciences, University of Rwanda (No: 233/CMHS IRB/2021). As the study relied solely on secondary data, informed consent from participants was not required. However, access to the dataset was granted after obtaining permission from the Emergency Medical Services (EMS) and data custodians who oversee records of patients. All data were anonymized prior to analysis to ensure confidentiality and privacy. All methods were conducted in accordance with the ethical standards outlined in the Declaration of Helsinki. ^38^

## Results

### Descriptive analysis

The participants of this study had a mean age of 31.64 years (SD = 11.1), with the largest age group (38%) falling between 20 and 29 years, highlighting a high burden of RTIs among young adults. A substantial majority (78.4%) were male, and over half (55.9%) were uninsured at the time of injury. Most collisions occurred in the afternoon (2:00 pm–7:59 pm; 28.9%), followed closely by morning to early afternoon incidents (8:00 am–1:59 pm; 27.1%), indicating peak risk during active daytime hours. Alcohol use was reported in 24.5% of cases, while 54.9% of participants had no history of alcohol users, pointing to behavioral risk factors among a considerable segment of the population. Regarding clinical presentation, extremity injuries were the most common (46%), followed by TBI at 22.2%, indicating high prevalence of both types. On average, individuals traveled 15.8 kilometers (SD=12.45) to access health services, with 68.8% residing within a 0–20 km radius, suggesting moderate geographic access to care ( Table 1).

**Table 1.** Description of the participants and all variables N=1162.

Variables	Frequency	%age
**Gender**		
Males	911	78.4
Females	251	21.6
**Age of participants**		
<20 years	107	9.2
20-29 years	442	38.0
30-39 years	390	33.6
40-49 years	153	13.2
50 years and above	70	6.0
**Health insurance**		
No	649	55.9
Yes	513	44.1
**Time of collision**		
8:00 am to 1:59 pm	315	27.1
2:00 pm to 7:59 pm	336	28.9
8:00 pm to 1:59 am	292	25.1
2:00 am to 7:59 am	219	18.8
**Chief complaints**		
TBI/head injury	258	22.2
Extremity injury	535	46
Chest pain	48	4.1
Abdominal pain/vomiting or nausea	15	1.3
Altered mental status	20	1.7
Other	286	24.6
**Trauma mechanism**		
Car only	47	4
Moto to moto	150	12.9
Bike only	23	2.0
Car to pedestrian	124	10.7
Car to moto	358	30.8
Moto only	63	5.4
Bike to pedestrian	215	18.5
Car to car	32	2.8
Bike to moto	80	6.9
Bike to car	70	6.0
**Substance use**		
No	638	54.9
Yes	285	24.5
Unknown	239	20.6
**Distance**		
0-20 km	799	68.8
21-40 km	334	28.7
More than 40 km	29	2.5

### Prevalence of severe injury by demographic and clinical information

**Figure 1.**
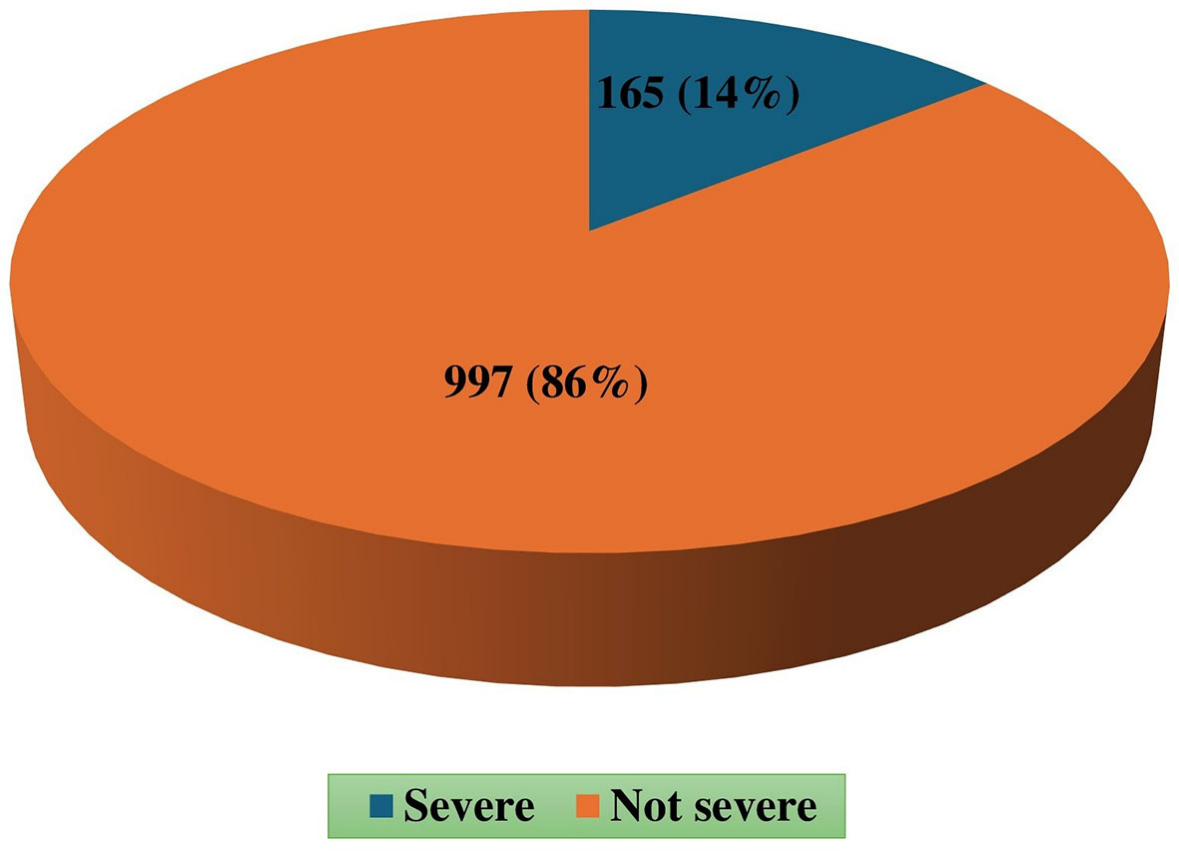
Prevalence of severe trauma.

A total of 1162 victims were considered that the prevalence of severe injury was 14% (n=165), with males accounting for 82.4% of these cases ( Figure 1).

The highest prevalence was observed among individuals aged 30–39 years (35.8%) and 20–29 years (35.2%). Individuals without health insurance had a significantly higher prevalence of severe injury (61.2%). RTIs occurring between 8:00am and 1:59pm had the highest prevalence of severe injury (38.2%). Extremity injuries were the most common severe complaint (40.6%), followed by traumatic brain injuries (35.8%). Those who were not alcohol users had a slightly higher prevalence of severe injury compared to those who used (35.2% vs 31.5%). Among the mechanisms of injury, car-to-motorcycle collisions had the highest prevalence of severe injury (31.5%) ( Table 2).

**Table 2.** Prevalence of severe injury by demographics and clinical characteristics (N=1162).

Variables	Severe injury		Not severe injury		
	N	%	N	%	Total
**A. Socio-demographic characteristics**					
**Gender**					
Males	136	82.4	775	77.7	911
Females	29	17.6	222	22.3	251
**Age of participants**					
<20 years	18	10.9	89	8.9	107
20-29 years	58	35.2	384	38.5	442
30-39 years	59	35.8	331	33.2	390
40-49 years	18	10.9	135	13.5	153
50 years and above	12	7.3	58	5.8	70
**Health insurance**					
No	101	61.2	548	55.0	649
Yes	64	38.8	449	45.0	513
**Time of collision**					
8 am to 1:59 pm	63	38.2	268	26.9	331
2 pm to 7:59 pm	41	24.8	296	29.7	337
8 pm to 1:59 am	40	24.2	237	23.8	277
2 am to 7:59 am	21	12.7	196	19.7	217
**Primary complaints**					
TBI/head injury	59	35.8	199	20.0	258
Extremity injury	67	40.6	468	46.9	535
Chest pain	3	1.8	45	4.5	48
Abdominal pain/vomiting or nausea	1	0.6	14	1.4	15
Altered mental status	12	7.3	8	0.8	20
Other	23	13.9	263	26.4	286
**Trauma mechanism**					
Car only	5	3.0	42	4.2	47
Moto to motor	15	9.1	135	13.5	150
Bike only	1	0.6	22	2.2	23
Car to pedestrian	30	18.2	94	9.4	124
Car to motor	52	31.5	306	30.7	358
Moto only	10	6.1	53	5.3	63
Bike to pedestrian	20	12.1	195	19.6	215
Car to car	5	3.0	27	2.7	32
Bike to motor	9	5.5	71	7.1	80
Bike to car	18	10.9	52	5.2	70
**Alcohol users**					
No	58	35.2	580	58.2	638
Yes	52	31.5	233	23.4	285
Unknown	55	33.3	184	18.5	239
**Distance**					
0-20 km	108	65.5	691	69.3	799
21-40 km	45	27.3	282	28.3	327
More than 40 km	12	7.3	24	2.4	36

### Bivariate and multivariate logistic regression analyses for the associated factors of severe injury

The findings from bivariate logistic regression analysis demonstrated that gender time of collision, primary complaints, trauma mechanism, alcohol use, and distance to the treating health facility were significant factors of severe injury among the pre-hospital emergency patients. All significant variables in bivariate logistic regressions were transported into multivariate logistic regression models. After adjusting for other variables, females had lower odds to experience severe injury compared to males (aOR=0.47, 95%CI: 0.26–0.79, p=0.032). In terms of trauma mechanism, we found that car-to-pedestrian collisions had more than twice the odds of severe injury (aOR=2.3, 95% CI: 1.25–4.1, p=0.007) compared to car-only accidents. Furthermore, car-to-motorcycle collisions posed an even greater risk, with nearly four times the odds of experiencing severe injury (aOR=3.88, 95% CI: 1.16–13.05, p=0.007) relative to car-only crashes. Alcohol use was also a strong predictor of severe injury, with alcohol users being more than three times as likely to experience severe injuries compared to non-users (aOR=3.37, 95% CI: 2.04–5.56, p<0.001). Regarding the timing of collisions, individuals involved in RTIs between 2:00 pm and 7:59 pm (aOR=0.52, 95% CI: 0.25–0.72, p=0.02) and 8:00 pm to 1:59 am (aOR=0.25, 95%CI: 0.04–0.81, p =0.012) were significantly less likely to experience severe injury compared to those involved in RTIs between 8:00 am and 1:59 pm. Additionally, the distance to healthcare facilities played a critical role in injury severity, with individuals traveling between 21 and 40 km after an RTI having nearly three times the odds of severe injury (aOR=2.91, 95%CI: 1.27–6.63, p=0.011), and those traveling over 40 km facing a similarly elevated risk (aOR=2.64, 95% CI: 1.11–6.25, p=0.028) compared to those within 0–20 km of a healthcare facility. These findings highlight the strong associations between severe injury and collision type, alcohol use, time of occurrence, and access to healthcare, underscoring the urgent need for targeted trauma prevention strategies to address these risk factors. In addition, the individuals who sustained extremity injuries (aOR=0.28, 95% CI: 0.15–0.52, p<.001), chest injuries (aOR=0.40, 95% CI: 0.23–0.71, p=.002), or other types of injuries (aOR=0.16, 95% CI: 0.05–0.57, p=.004) were significantly less likely to suffer from severe injury compared to those with TBI or head trauma ( Table 3).

**Table 3.** Bivariate and multivariate logistic regression analyses of factors associated with severe injury among pre-hospital emergency patients (n=1162).

Variables		Bivariate analysis; 95% CI			p-value	Multivariate analysis, 95% CI			p-value
	N (%)	COR	Lower bound	Upper bound		aOR	Lower bound	Upper bound	
**A. Socio-demographic characteristics**									
**Gender**									
Males	911 (78.4)	1				1			
Females	251 (21.6)	0.61	0.21	0.94	0.002 **	0.47	0.26	0.79	0.036 *
**Age of participants**									
<20 years	107 (9.2	1			0.639				
20-29 years	442 (38)	1.02	0.46	2.28	0.956				
30-39 years	390 (33.6)	1.37	0.69	2.7	0.365				
40-49 years	153 (13.2)	1.16	0.59	2.29	0.668				
50 years and above	70 (6)	1.55	0.7	3.43	0.277				
**B. Clinical or health related characteristics**									
**Health insurance**									
No	649 (55.9)	1							
Yes	513 (44.1)	0.77	0.55	1.08	0.135				
**Time of collision**									
8:00 am to 1:59 pm	315 (27.1)	1			0.012 *	1			
2:00 pm to 7:59 pm	336 (28.9)	0.46	0.27	0.77	0.003 **	0.52	0.25	0.72	0.02 *
8:00 pm to 1:59 am	292(25.1)	0.77	0.44	1.35	0.365	0.25	0.04	0.81	0.012 *
2:00 am to 7:59 am	219 (18.8)	0.64	0.36	1.11	0.112	0.57	0.12	2.18	0.331
**Chief complaints**									
TBI/head injury	258 (22.2)	1			<0.001 ***	1			<.001 ***
Extremity injury	535 (46)	0.3	0.18	0.49	<0.001 ***	0.28	0.15	0.52	<.001 ***
Chest pain	48 (4.1)	0.61	0.37	1	0.052	0.4	0.23	0.71	.002 **
Abdominal pain/vomiting/nausea	15 (1.3)	1.31	0.38	4.55	0.669	0.85	0.23	3.1	0.803
Altered mental status	20 (1.7)	1.22	0.15	9.73	0.848	1.3	0.13	12.7	0.824
Other	286 (24.6)	0.06	0.02	0.16	<0.001 ***	0.16	0.05	0.57	.004 **
**Trauma mechanism**									
Car only	47 (4)	1			0.002 **	1			.011 *
Moto to motor	150 (12.9)	2.91	1	8.49	0.051	1.84	0.52	6.52	0.346
Bike only	23 (2)	3.12	1.46	6.64	0.003 **	1.78	0.71	4.47	0.223
Car to pedestrian	124 (10.7)	7.62	0.96	60.62	0.055	2.3	1.25	4.1	.007 **
Car to motor	358 (30.8)	1.08	0.55	2.13	0.814	3.88	1.16	13.05	.024 *
Moto only	63 (5.4)	2.04	1.11	3.75	0.023 *	1.06	0.49	2.29	0.73
Bike to pedestrian	215 (18.5)	1.83	0.77	4.35	0.168	1.15	0.4	3.32	0.839
Car to car	32 (2.8)	3.38	1.67	6.84	<0.001 ***	2.07	0.87	4.93	0.102
Bike to motor	80 (6.9)	1.87	0.63	5.58	0.263	1	0.29	3.48	0.998
Bike to car	70 (6)	2.73	1.14	6.56	0.025 *	1.3	0.47	3.68	0.611
**Alcohol users**									
No	638 (54.9)	1			<0.001 ***	1			<.001 ***
Yes	285 (24.5)	2.99	2	4.48	<0.001 ***	3.37	2.04	5.56	<.001 ***
Unknown	239 (20.6)	1.34	0.88	2.05	0.178	1.55	0.91	2.64	0.111
**Distance to the treating health facility**									
0-20 km	799 (68.8)	1			0.006 **	1			.04 *
21-40 km	334 (28.7)	3.2	1.55	6.59	0.002 **	2.91	1.27	6.63	.011 *
More than 40 km	29 (2.5)	3.13	1.46	6.71	0.003 **	2.64	1.11	6.25	.028 *

N: Frequency; 5: Percentage; CI: Confidence intervals; OR: Odds ratio.

* Emboldened values represent statistically significant at p<0.05.

** Emboldened values represent statistically significant at p<0.01.

*** Indicated the variables statistical significance at p<0.001.

## Discussion

This study aimed to assess the prevalence of severe injury and its associated factors among RTI victims who received pre-hospital care. Our findings revealed that 14% of cases involved severe injuries, which is comparable to studies conducted in Australia (10%), Ethiopia (13.8%), and Kenya (19%). ^1, 39– 41^ However, other studies, particularly in Tanzania and Ethiopia, reported a significantly higher prevalence, exceeding 36.4%. ^20^ These variations can be explained by differences in study methodologies. Our research relied on secondary data analysis and trauma registries from pre-hospital emergency care services, which primarily respond to RTI emergencies. The role of SAMU includes rescuing, providing immediate care, and transporting severely injured individuals to health facilities, mainly in Kigali, based on injury severity.

We found that younger individuals were not significantly more likely to suffer from severe injury, which contrasts with previous research ^40, 42^ that identified youth as a high-risk group due to factors such as risk-taking, inexperience, and unsafe driving behaviors. Despite not being a predictor of severe injury in our analysis, young adults (ages 20–39) still accounted for around 70% of all RTI cases, highlighting their greater involvement in traffic incidents overall. This underscores their vulnerability and the need for targeted preventive efforts. Additionally, consistent with earlier studies, ^40, 41, 43^ our findings confirmed that females had a lower likelihood of sustaining severe injuries compared to males, likely due to men’s increased exposure to high-risk driving situations and their higher participation in professional driving.

Our multivariable logistic regression analysis showed that collisions involving cars and pedestrians or cars and motorcyclists were associated with significantly higher odds of severe injury compared to car-only crashes. This aligns with existing literature indicating that vulnerable road users, such as pedestrians and motorcyclists, face increased odds of severe injury, which may be partly explained by their limited physical protection and greater exposure to traffic hazards. ^16, 44, 45^ These increased odds could be attributed to the insufficient or lack of physical protection for pedestrians, cyclists, and motorcyclists, which makes them more susceptible to severe injuries in road traffic incidents. Additionally, inadequate knowledge of road traffic rules among some vehicle users, coupled with frequent traffic violations and discourteous behavior, may further increase the odds of accidents. Consistent with prior studies, ^11^ we also found that alcohol use was associated with nearly threefold higher odds of sustaining severe injuries to themselves or others compared to sober drivers. These results reinforce the evidence that alcohol use substantially increases the odds of severe injury. ^46– 48^

Our study identified that road traffic injuries occurring between 2:00 pm and 1:59 am (afternoon to early night) were less likely to result in severe injury compared to those between 8:00 am and 1:59 pm (morning to early afternoon). This finding contrasts with several studies from sub-Saharan Africa, which report higher injury severity during evening and night hours. For instance, a study in Thika, Kenya, found that night-time crashes were associated with increased injury severity. ^41^ Similarly, research in Adama, Ethiopia, indicated that night-time injuries significantly elevated the risk of death. These findings align with a study from the Oromia region of Ethiopia, which reported fewer fatalities during evening and night hours compared to the afternoon. ^15, 49^ These discrepancies between our results and the previous studies may stem from differences in traffic volume, road user behavior, and environmental conditions across regions. In our context, higher traffic density during morning hours could contribute to increased injury severity. In contrast, in settings where night-time driving is more hazardous due to factors like poor visibility or inadequate lighting, evening and night crashes may result in more severe outcomes. Therefore, contextual factors such as local traffic patterns, infrastructure, and enforcement of road safety measures likely influence the relationship between time of day and injury severity. ^49, 50^

The study demonstrated that individuals presenting with extremity injuries had a lower likelihood of experiencing severe injury compared to those with TBI or head trauma. This distinction is clinically significant, as injuries to the brain, neck, and spinal cord often involve vital structures and can lead to rapid deterioration, long-term disability, or death—even when external signs may appear less dramatic than in cases of open fractures or limb trauma. These findings are consistent with a previous study that reported head, neck and spinal cord injury as the major factors of severe injury. ^1^ Additionally, a greater distance to a hospital increased the likelihood of sustaining severe injuries, with individuals traveling 21-40 km and those traveling more than 40 km being determinants of severe injuries compared to those traveling less than 21 km for definitive healthcare services. This likely reflects both delays in reaching care and potential deterioration during transit especially in contexts with limited prehospital stabilization and transport infrastructure. These results collaborate with the previous studies that reported the long distance is a risk factor of severe injury. ^42, 51^

### Study strengths and limitations

This study has some notable strengths. It utilized a large registry dataset covering pre-hospital care in Kigali, which enhances statistical power and supports broader generalization of the findings. Additionally, this study addresses a less-researched topic in Rwanda, shedding light on a significant public health issue that necessitates the implementation of targeted strategies. The insights gained from this study can inform policy decisions and interventions aimed at reducing the burden of road traffic injuries and improving road safety measures.

Despite its strengths, this study has several limitations that warrant discussion. Firstly, the use of secondary data from registries restricted our ability to include certain key predictors, such as pre-hospital care details (e.g. care provided at the scene, mode of transport to the hospital, use of spinal immobilization or trauma protocols), socio-demographic factors (e.g., marital status, education, employment status, and type of residence), and hospital-related variables (such as length of hospital stay, surgical interventions). Additionally, safety-related variables, such as adherence to traffic regulations, data on safety device usage, seatbelt and helmet utilization, and the duration of possessing a driver’s license before the accident, were not documented, limiting our understanding of factors influencing injury severity. Secondly, environmental factors, including road type and accident location, were not captured, which may have influenced injury outcomes. Moreover, the classification of distance to the health facility was not based on established evidence, which might have introduced bias through possible over- or underestimation. Finally, the retrospective cross-sectional design of the study prevents the establishment of causal relationships between the identified predictors and injury severity.

### Public health implications

The findings of this study highlight an urgent need for public health interventions to address road RTIs and their associated consequences. The high prevalence of severe injury among alcohol users reinforces the importance of targeted awareness campaigns focused on reducing alcohol and alcohol use, particularly among young male drivers, who represented a significant proportion of severe cases. Behavioral change programs tailored for this demographic could play a critical role in reducing high-risk behaviors on the road. Moreover, the elevated risk of severe injury in pedestrian and motorcycle-related collisions underscores the vulnerability of these road users. Policy interventions such as creating pedestrian-friendly infrastructure, implementing protected lanes for cyclists and motorcyclists, and enforcing speed reduction zones in high-traffic areas are essential to safeguard these populations. The study also found that injury severity was influenced by the time of day, suggesting a need for improved traffic regulation and law enforcement during high-risk periods, particularly in the early morning and evening hours. Finally, access to timely medical care remains a critical issue, especially for those living in rural or remote areas. Strengthening pre-hospital emergency systems and decentralizing trauma care services to underserved regions will be key to improving trauma outcomes and reducing preventable deaths. These findings highlight the importance of prioritizing high-risk patients within emergency medical services and developing targeted prevention and trauma care strategies to improve patient outcomes and reduce the overall burden of severe injuries. These implications call for an integrated public health approach that combines education, policy enforcement, and infrastructure development. By prioritizing these measures, policymakers and stakeholders can make significant strides in reducing the burden of RTIs and promoting safer road environments.

### Future directions

To expand upon the findings of this study, future research should prioritize longitudinal and prospective designs to establish causal links between various risk factors and severe injury outcomes. A more comprehensive investigation of socio-demographic and environmental factors will offer deeper insights into their influence on road traffic injuries. Additionally, exploring healthcare-related aspects including hospital-specific factors could enhance understanding of their effects on injury severity, recovery, and disability.

Given the limited understanding of how prehospital emergency care influences trauma severity, the integration of innovative methodologies like participatory action research (PAR) could prove particularly valuable in preventing RTIs and their outcomes. ^52^ By actively engaging community members, stakeholders, and policymakers throughout the research process, PAR fosters a collaborative exploration of local challenges and facilitates the co-creation of interventions aiming sustainable changes especially reducing RTIs. Moreover, employing mixed methods in future research can provide a holistic understanding of the complexities surrounding road traffic injuries. While quantitative data can reveal statistical trends in injury prevalence and associated factors, qualitative methods can uncover the lived experiences, perceptions, and behaviors of road users. This integrative approach allows for a nuanced analysis of underlying issues and barriers to road safety, informing more effective public health interventions. Lastly, collaborative efforts among public health officials, policymakers, and community stakeholders are essential for devising and implementing strategies to reduce road traffic injuries and promote safer road practices. By incorporating PAR and mixed methods, future research can not only guide policy development but also empower road users to actively participate in creating safer environments.

## Conclusion

This study identified several factors associated with severe injury, including traveling distance to emergency services, gender, alcohol use, time of day, injury type, and mechanisms of trauma. These findings highlight the importance of targeted and context-specific interventions to reduce the risk of severe injury. Improving access to prehospital emergency care, enforcing regulations on alcohol use, and promoting safer road behaviors are practical measures that could enhance injury prevention efforts. Additionally, strengthening trauma care systems and data collection practices particularly in resource-limited settings can support more timely and effective emergency responses. Clinically, the identified predictors of severe injury can support improved triage and risk stratification in emergency departments. Incorporating these factors into clinical assessment tools may help providers rapidly identify and prioritize high-risk patients, especially in resource-constrained settings where timely intervention is critical. Future research, particularly using longitudinal designs, is recommended to further investigate causal relationships and inform evidence-based policy and clinical practice.

## Data Availability

The data analyzed and reported in this study are fully included in the manuscript. Additional datasets used or generated during the study can be obtained from the corresponding author upon reasonable request. The dataset can be accessed via https://doi.org/10.5281/zenodo.17489912. ^53^
