# Supplementary material for: Factors associated with road traffic injury severity among victims retrieved by pre-hospital emergency services in Rwanda
Source: F1000Res. 2026 Feb 5;14:1282. Originally published 2025 Nov 19. [Version 2] doi: 10.12688/f1000research.172231.2 (PMC13157606; doi:10.12688/f1000research.172231.2)
Supplement: Peer review history

# Reviewer report: Reviewer response for version 2

**DOI:** 10.5256/f1000research.195770.r481760

**Published:** 2026-05-09

**Authors:** Muhammed Navid Tahir

**Affiliations:** 1 University of the Punjab, Lahore, Pakistan

Though study does not have novel idea/subject yet it has some strenghts such as it is originated from destination where there is limited research especially in road safety and injury prevention area. Moreover authors have strengthened methodology part, and provided a more explicit justification of key methodological choices, including the injury severity threshold (ISS >9), the selection of predictive variables, and the categorization of distance to care. Therefore this version of manuscript offers improved methodological and interpretative rigor. Study also provides public health implications

Is the work clearly and accurately presented and does it cite the current literature?

Yes

If applicable, is the statistical analysis and its interpretation appropriate?

Yes

Are all the source data underlying the results available to ensure full reproducibility?

Yes

Is the study design appropriate and is the work technically sound?

Yes

Are the conclusions drawn adequately supported by the results?

Yes

Are sufficient details of methods and analysis provided to allow replication by others?

Yes

Reviewer Expertise:

road safety, injury prevention, public health, prevention of non-communicable diseases, pre-hospital care,

I confirm that I have read this submission and believe that I have an appropriate level of expertise to confirm that it is of an acceptable scientific standard.

# Reviewer report: Reviewer response for version 2

**DOI:** 10.5256/f1000research.195770.r455827

**Published:** 2026-02-20

**Authors:** Emmanuel Bonnet

**Affiliations:** 1 Institut de Recherche pour le Développement, CNRS Université Paris 1 Panthéon- Sorbonne, Île-de-France, France

I thank the authors for their careful and detailed responses to the comments previously raised. The revised version has improved in clarity and methodological transparency.

 The authors have provided satisfactory elements regarding the high exclusion rate by acknowledging its potential implications and contextualizing pre-hospital data collection in Rwanda. Although residual bias cannot be entirely ruled out, this limitation is now explicitly discussed.

 The justification for the ISS > 9 threshold is better argued and more clearly situated within the context of pre-hospital care and low-resource settings, which strengthens the coherence of the severity classification.

 The clarifications provided regarding the use of chief complaints as predictive variables are acceptable, particularly from a pre-hospital triage perspective. The methodological choices are now sufficiently explained.

 The categorization of distance variables and the assessment of alcohol consumption have also been clarified; this remains not entirely satisfactory, but I believe it is unlikely that further improvement is possible at this stage.

 Overall, the authors have responded constructively to the comments, and the manuscript has improved accordingly. At this stage, I consider that the concerns raised have been appropriately addressed and that it is probably not possible to go further given the available data.

Is the work clearly and accurately presented and does it cite the current literature?

Yes

If applicable, is the statistical analysis and its interpretation appropriate?

Yes

Are all the source data underlying the results available to ensure full reproducibility?

Yes

Is the study design appropriate and is the work technically sound?

Yes

Are the conclusions drawn adequately supported by the results?

Yes

Are sufficient details of methods and analysis provided to allow replication by others?

Yes

Reviewer Expertise:

Health geographer

I confirm that I have read this submission and believe that I have an appropriate level of expertise to confirm that it is of an acceptable scientific standard.

# Reviewer report: Reviewer response for version 1

**DOI:** 10.5256/f1000research.189946.r434978

**Published:** 2025-12-04

**Authors:** Emmanuel Bonnet

**Affiliations:** 1 Institut de Recherche pour le Développement, CNRS Université Paris 1 Panthéon- Sorbonne, Île-de-France, France

 This article examines the factors associated with the severity of road traffic injuries (RTIs) among victims managed by the SAMU (Emergency Medical Service) in Rwanda in 2020. The study is relevant as it addresses a major public health issue in a Low- and Middle-Income Country (LMIC), focusing on the pre-hospital phase, which is often under-researched.

 However, there are elements that need clarification and improvement, notably a potential selection bias (high exclusion rate) and debatable choices regarding predictive variables. These points must be addressed before full approval.

 Regarding the methodological aspects: The use of the SAMU electronic registry is a sound approach to obtaining real-world clinical data and is particularly valuable given its rarity on the continent. The use of the Injury Severity Score (ISS) to classify severity is also suitable for the African context. However, I have a few questions/concerns:

1. The authors indicate that out of 2,062 identified cases, 900 were excluded due to missing data, leaving only 1,162 patients for analysis. This represents an exclusion rate of nearly 44%. It is necessary to specify whether this missing data is random or not. Could patients who died before arrival or those with very severe injuries be overrepresented in the missing data? The urgency of care could explain the incompleteness of the information. The authors must compare, if possible, the baseline characteristics of the excluded vs. included patients, or at least discuss this bias more deeply in the limitations section. Otherwise, there is a significant selection bias.

- 2. The study dichotomizes severity at an ISS > 9. Although justified by the authors, an ISS of 9 is often considered "moderate." A threshold of 15 (major trauma) is more common in international literature to define a "severe injury." The authors should further justify this choice by citing references such as: *Copes WS, Champion HR, Sacco WJ, Lawnick MM, Keast SL, Bain LW. "The Injury Severity Score revisited." The Journal of Trauma. 1988;28(1):69-77.*
- 3. In the logistic regression (Table 3), the authors include "Chief complaints" (e.g., TBI/head injury, Extremity injury) as independent variables to predict "Severity." I find this surprising because, inherently, a TBI predicts a high score. The ISS is calculated based on anatomical injuries. The authors need to provide further justification for their choice of independent variables.
- 4. The correlation between a distance >20km and severity is a very interesting result. However, the categorization (0-20, 21-40, >40) seems arbitrary, as mentioned in the limitations; would it be possible to modify this to allow for a finer analysis?
- 5.Regarding alcohol, it should be specified how consumption was assessed.
- 6. In the limitations section, the issue regarding missing data mentioned above must be detailed.

Is the work clearly and accurately presented and does it cite the current literature?

Yes

If applicable, is the statistical analysis and its interpretation appropriate?

Yes

Are all the source data underlying the results available to ensure full reproducibility?

Yes

Is the study design appropriate and is the work technically sound?

Yes

Are the conclusions drawn adequately supported by the results?

Yes

Are sufficient details of methods and analysis provided to allow replication by others?

Yes

Reviewer Expertise:

Health geographer

I confirm that I have read this submission and believe that I have an appropriate level of expertise to confirm that it is of an acceptable scientific standard, however I have significant reservations, as outlined above.

# Response

**Published:** 2026-01-25

**Authors:** Eric Uwitonze

**Affiliations:** School of Public Health, University of Rwanda College of Medicine and Health Sciences, Kigali, Kigali City, Rwanda

**Report for reviewers**

 This article examines the factors associated with the severity of road traffic injuries (RTIs) among victims managed by the SAMU (Emergency Medical Service) in Rwanda in 2020. The study is relevant as it addresses a major public health issue in a Low- and Middle-Income Country (LMIC), focusing on the pre-hospital phase, which is often under-researched.

 However, there are elements that need clarification and improvement, notably a potential selection bias (high exclusion rate) and debatable choices regarding predictive variables. These points must be addressed before full approval.

**Answer:** We thank the reviewer for the careful assessment of our manuscript and for raising important methodological concerns. Regarding the high exclusion rate and potential selection bias, we acknowledge this as a key limitation of our study. The SAMU data management system in Rwanda in 2020 was still evolving and was not sufficiently robust to ensure complete and systematic recording of all clinically relevant variables. Consequently, a considerable number of records contained missing or incomplete information on essential variables, which led to their exclusion from the analysis. This situation is understandable in a pre-hospital emergency context in a low-resource setting, where the priority is rapid life-saving care rather than detailed data documentation.

 This challenge is not unique to our study. Previous research has similarly reported limitations in the use of SAMU datasets due to incomplete and inconsistent reporting, which has restricted broader use of these data for research purposes. Our study therefore represents one of the few attempts to analyze RTI severity using SAMU data despite these constraints.

 To minimize bias, exclusions were based strictly on objective criteria related to data completeness, rather than on injury severity or patient characteristics. While we cannot entirely rule out selection bias, there is no indication that excluded cases differed systematically from included cases in a way that would invalidate the observed associations. We have explicitly acknowledged this limitation in the manuscript and advised caution in interpreting and generalizing the findings.

 Regarding the selection of predictive variables, our choices were guided by (1) the consistent availability of variables in the SAMU database, (2) their clinical and operational relevance in the pre-hospital setting, and (3) evidence from the existing literature on road traffic injuries in LMICs. Although additional variables could potentially improve prediction of injury severity, they were not reliably recorded, and their inclusion would have introduced further bias.

 Finally, by explicitly recognizing these data limitations, we have translated them into a practical recommendation in the manuscript. We emphasize the need for improved and standardized reporting of all cases managed by SAMU, including more complete documentation of clinical and contextual variables. Strengthening routine EMS data collection will not only enhance patient care and system monitoring but also enable higher-quality research and more robust evidence to inform injury prevention and emergency care policies in Rwanda.

 Regarding the methodological aspects: The use of the SAMU electronic registry is a sound approach to obtaining real-world clinical data and is particularly valuable given its rarity on the continent. The use of the Injury Severity Score (ISS) to classify severity is also suitable for the African context. However, I have a few questions/concerns:

 1. The authors indicate that out of 2,062 identified cases, 900 were excluded due to missing data, leaving only 1,162 patients for analysis. This represents an exclusion rate of nearly 44%. It is necessary to specify whether this missing data is random or not. Could patients who died before arrival or those with very severe injuries be overrepresented in the missing data? The urgency of care could explain the incompleteness of the information. The authors must compare, if possible, the baseline characteristics of the excluded vs. included patients, or at least discuss this bias more deeply in the limitations section. Otherwise, there is a significant selection bias.

**Answer:** We acknowledge the high exclusion rate as an important limitation. Missing data largely reflect the operational realities of pre-hospital emergency care in Rwanda, where documentation may be incomplete in high-urgency cases. The SAMU registry does not systematically capture pre-hospital outcomes or allow formal assessment of whether missingness was random, limiting comparisons between excluded and included patients. Exclusions were based solely on objective data completeness criteria. We now discuss the potential for non-random missingness and selection bias more explicitly in the Limitations section and recommend improved standardized reporting within SAMU.

 2. The study dichotomizes severity at an ISS > 9. Although justified by the authors, an ISS of 9 is often considered "moderate." A threshold of 15 (major trauma) is more common in international literature to define a "severe injury." The authors should further justify this choice by citing references such as: *Copes WS, Champion HR, Sacco WJ, Lawnick MM, Keast SL, Bain LW. "The Injury Severity Score revisited." The Journal of Trauma. 1988;28(1):69-77.*

**Answer:** We thank the reviewer for this important methodological point. We acknowledge that ISS 15 is commonly used in international literature to define major trauma, particularly in high-income hospital-based trauma registries (e.g., Copes et al., 1988). However, in the pre-hospital context and LMIC settings, lower ISS thresholds (ISS >9) have frequently been used to distinguish clinically meaningful moderate-to-severe injuries requiring urgent care, referral, or advanced management. In Rwanda’s SAMU setting, patients with ISS >9 already represent a group with substantially higher clinical acuity and resource needs. Moreover, using ISS ≥15 would have resulted in a small subgroup, limiting statistical power and interpretability. We have clarified and strengthened this justification in the Methods and Discussion sections, while acknowledging this choice as a contextual consideration. To do this classification, we referred to the local evidence/national standards that showed those with more than 9 ISS have severe injuries. We found there are many studies that contrast the classifications of Copes et al. (1988). See the following studies used as references:

1. Traisathit, P., Chittawatanarat, K., Chandacham, K. et al. Trauma referral audit impact assessment on the outcomes of injured patients via an interrupted time-series analysis: an 11-year before-and-after study of trauma cases at the Maharaj Nakorn Chiang Mai hospital, Thailand. BMC Emerg Med 25, 64 (2025). https://doi.org/10.1186/s12873-025-01220-0)
2. Abajas-Bustillo R, Amo-Setién FJ, Leal-Costa C, Ortego-Mate MDC, Seguí-Gómez M, Durá-Ros MJ, Zonfrillo MR. Comparison of injury severity scores (ISS) obtained by manual coding versus "Two-step conversion" from ICD-9-CM. PLoS One. 2019 May 1;14(5):e0216206. https://doi.org/10.1371/journal.pone.0216206.
3. Reith, G., Lefering, R., Wafaisade, A. et al. Injury pattern, outcome and characteristics of severely injured pedestrian. Scand J Trauma Resusc Emerg Med 23, 56 (2015). https://doi.org/10.1186/s13049-015-0137-8
  (These support the use of ISS >9 to distinguish clinically important injury severity levels when conventional major trauma cutoffs (e.g., ISS ≥15 or ≥16) may be less practical due to sample size or context, especially in pre-hospital and LMIC RTI research).

3.In the logistic regression (Table 3), the authors include "Chief complaints" (e.g., TBI/head injury, Extremity injury) as independent variables to predict "Severity." I find this surprising because, inherently, a TBI predicts a high score. The ISS is calculated based on anatomical injuries. The authors need to provide further justification for their choice of independent variables.

**Answer**: We thank the reviewer for this insightful comment. We acknowledge that ISS is anatomically based and that certain injuries, such as TBI, inherently contribute to higher ISS scores. However, in the pre-hospital EMS context, “chief complaints” recorded by SAMU reflect the initial clinical presentation before detailed anatomical scoring is available. Including these variables allows us to examine which pre-hospital observable injuries or complaints are associated with higher eventual ISS, providing insight into early triage and resource allocation. We have clarified this rationale in the Methods section and emphasized that these variables represent pre-hospital indicators rather than post-hoc predictors, highlighting their operational relevance.

 Predictive variables were selected based on their consistent availability in the SAMU registry, clinical relevance in the pre-hospital setting, and prior literature on road traffic injury severity in LMICs. Variables not consistently recorded were excluded to maintain analytical rigor and reduce bias.

 4. The correlation between a distance >20km and severity is a very interesting result. However, the categorization (0-20, 21-40, >40) seems arbitrary, as mentioned in the limitations; would it be possible to modify this to allow for a finer analysis?

**Answer:** We thank the reviewer for highlighting this important finding. We acknowledge that these cutoffs may appear arbitrary and limit granularity. Unfortunately, distance was not consistently recorded as a continuous variable, which precluded a finer or alternative categorization (this is because our dependent variables are also categorical variable). Distances were grouped into three categories (0–20, 21–40, >40 km) informed by the distribution of SAMU data and national geographic and service delivery considerations; although prior studies did not categorize distance, these groupings were designed to capture meaningful access challenges, as distances beyond short travel ranges are known to delay care and align with national objectives to improve timely access to emergency services.

 5.Regarding alcohol, it should be specified how consumption was assessed.

**Answer:** Alcohol consumption was assessed based on information recorded in the SAMU registry at first patient contact, including patient self-report or observation by the attending EMS personnel (e.g., smell of alcohol or reported recent intake). However, SAMU has standardized instruments (alcohol test) used to measure the level of alcohol. We have clarified this assessment method in the Methods section (see page 4, subsection of study variables).

 6. In the limitations section, the issue regarding missing data mentioned above must be detailed.

**Answer**: We have expanded the Limitations section to provide a more detailed discussion of missing data, explaining its extent, likely causes related to the pre-hospital emergency context, the resulting exclusions, and the potential for non-random missingness and selection bias, as well as their implications for interpretation and generalizability of the findings (see page 16).
